# Effect of NADPH oxidase inhibitor apocynin on human lung cancer A549 cells via Bcl-2, Bax, caspase-3, and NF-κB signaling pathway

**DOI:** 10.1007/s00210-025-03833-5

**Published:** 2025-02-13

**Authors:** Betul Apaydın Yıldırım, Tuba Dogan, İsmail Bolat, Ali Can Ozcan, Rabia Kocak

**Affiliations:** 1 Department of Biochemistry, Faculty of Veterinary, Ataturk University, Erzurum, Türkiye; 2 Department of Pathology, Faculty of Veterinary, Ataturk University, Erzurum, Türkiye; 3 Department of Internal Medicine, Faculty of Veterinary, Ataturk University, Erzurum, Türkiye; 4 Department of Molecular Biology and Genetics, Faculty of Science, Ataturk University, Erzurum, Türkiye

**Keywords:** A549 cell, Apocynin, Apoptosis, Cytotoxicity, MTT, Oxidative stress

## Abstract

Apocynin (AP) is an anti-inflammatory drug with different therapeutic effects. This study aimed to investigate the antiproliferative, apoptotic, and antioxidant effects of AP on human lung adenocarcinoma cells (A549) and to investigate the effects on Bax, Bcl-2, NF-κB, and caspase-3 signaling pathways that may play a role in the pathogenesis of lung cancer. Cell viability was measured by MTT and cell apoptosis, and detection of cell death was measured by ELISA. IMA, AOPP, MDA, and GSH levels, SOD and CAT activities, and Bcl-2, Bax, NF-κB, and caspase-3 expression levels were analyzed from the obtained cell lysates. As a result, according to the findings obtained, IMA and MDA levels decreased in the A549 cancer cell line, while GSH levels and SOD and CAT activity increased. It was determined that the application of apocynin to A549 cells significantly reduced cell viability and directed the cells to apoptosis, increased Bax, NF-κB, and caspase-3 expression, and decreased Bcl-2 expressions. Since all of the data obtained were not found in the literature about the use of apocynin in the A549, the study conducted is pioneering. Our study demonstrates the potential of apocynin for cancer therapy possibly targeting the apoptotic pathway.

**Graphical Abstract:**

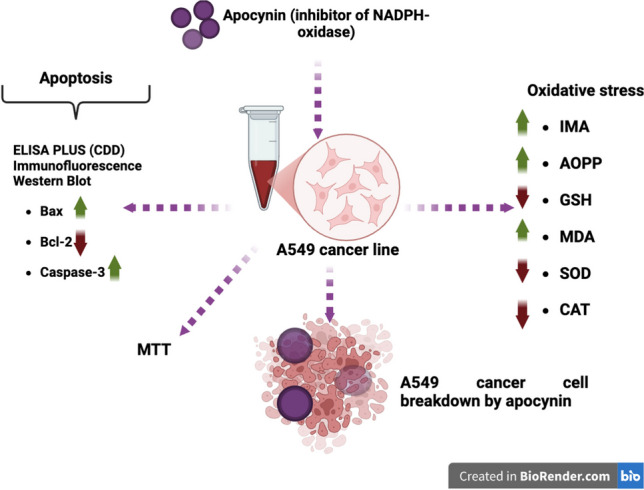

## Introduction

Lung cancer is the most common prevalent cancer globally, causing 1.6 million deaths. Its frequency has increased due to the introduction of smoking and marketing in new countries (Ferlay 2015, 2019). Approximately 75% of cancer deaths in women and men are due to lung cancer. Overexpression of many proteins leads to lung cancer metastasis (Chen et al. 2018).

Apocynin called 4‐hydroxy‐3‐methoxy‐acetophenone (AP) is extracted from *Picrorhiza kurroa* (Scrophulariaceae) roots and *Apocynum cannabinum* (Canadian hemp). It is a naturally occurring compound that contains a methoxy group and a catechol structure. This compound has the ability to selectively and specifically inhibit a specific enzyme called NOX in activated white blood cells, which prevents the production of a harmful substance called reactive oxygen species (ROS) molecules (Stefanska and Pawliczak 2008). Acetophenones, phenolic compounds found in fungal species and more than 24 plant families, occur in nature in both free and glycoside forms. Numerous biological activities, such as antibacterial, cytotoxic, antioxidant, antityrosinase, and antimalarial properties, have been discovered for these substances (Ahmadpourmir et al. 2024). In recent years, a natural, inexpensive molecule derived from the Himalayan medicinal plant *Picrorhiza kurroa* has been suggested as an anti-inflammatory, antioxidant, and anticarcinogenic agent because it can react directly with ROS molecules. By blocking the cytosolic p47phox subunit’s translocation and expression to the membrane, apocynin inhibits NADPH oxidase (NOX) (Vejražka et al. 2005; Hur et al. 2010).

An important role in pathological and physiological processes excessive production of reactive ROS causes oxidative stress, which accelerates cellular dysfunction, leading to various diseases including cancer (Franco et al. 2008), atherosclerosis (Ohashi et al. 2006), inflammatory bowel disease (Zhu and Robert 2012), and pulmonary inflammation (Kouki et al. 2023). NOX-derived ROS are particularly implicated in oxidative stress–related pathological conditions (Lambeth 2007). Numerous cell types experience apoptotic cell death due to ROS, and clinical disorders result from the dysregulation of apoptosis. Since ROS are extremely fragile, their production is usually controlled by a variety of substances known as antioxidant agents, including both nonenzymatic and enzymatic ones like catalase (CAT), superoxide dismutase (SOD), and glutathione peroxidase (GPx) (Datta et al. 2015; Endesfelder et al. 2019).

Mitochondrial molecules, the energy factories of the cell, play crucial roles in regulating cell death, signaling, and cellular differentiation. They influence apoptosis in various cell lines by contributing to ROS formation, cytochrome c release, caspase-3 and Bax activation, Bcl-2 downregulation, and the release of apoptosis-inducing factor (Efferth et al. 2007; Hamacher-Brady et al. 2011). Mitochondrial membrane permeability pores are reported to be redox-sensitive, with ROS facilitating their permeability. Dysregulated Bax and Bcl-2 levels impair mitochondrial function, leading to cytochrome c release into the cytosol and subsequent activation of caspase cascades, the final step in apoptosis (Li et al. 2010). Bax, a pro-apoptotic Bcl-2 family protein, is predominantly cytosolic in healthy cells. Upon death signal initiation, it oligomerizes and integrates into the outer mitochondrial membrane, facilitating the export of apoptogenic proteins.

According to recent research, AP attenuated lung injury by suppressing the NF-κB (nuclear factor-κB) pathway, lowering lipid peroxidation, and inhibiting NOX expression and NOX activity; therefore, the transcription of proinflammatory cytokines in lung tissue was reduced (Choi et al. 2017). AP also suppressed the production of proinflammatory cytokines, including interleukin-1β, TNF-α, and IL-6 (Kim et al. 2012). Numerous investigations have demonstrated the advantageous anti-apoptotic, antioxidant, and anti-inflammatory effects of AP in conditions affecting the lungs and airways, such as lung fibrosis, chronic obstructive pulmonary disease, and asthma (Wieczfinska et al. 2019; Wieczfinska et al. 2020). Several animal studies and cell culture studies have shown that AP can reduce neutrophil chemotaxis and neutrophil oxidative bursts, thereby reducing neutrophil‐mediated cell injury (Impellizzeri et al. 2011). Therefore, considering the antiproliferative, antioxidant, and apoptotic effects of AP, we aimed that AP could potentially have protective effects against lung cancer.

However, no literature has been found regarding the caspase-3, Bax, NF-κB, and Bcl-2 pathways of apocynin in the A549 cell line. This study aims to investigate apocynin’s anticancer properties against human lung cancer A549 cells as well as its mode of action.

## Materials and methods

### Materials

The lung human cancer (A549 cell line) cell line was bought from the ATCC (American Type of Culture Collection, Bethesda, USA, MD). We used Dulbecco’s Modified Eagle’s Medium (DMEM), fetal bovine serum (FBS, Cat No. 10099141), penicillin-streptomycin, and 2 mM L-glutamine (Cat No. 10378016) (Gibco^TM^, Thermo Fisher Scientific, Inc., Waltham, MA, USA). Thiazolyl blue tetrazolium bromide (MTT), DMSO, apocynin, and all powder chemicals were obtained from Sigma-Aldrich, St. Louis, MO, USA. RIPA (sc-24948), Bax (sc-20067), Bcl-2 (sc-7382), caspase-3 (sc-56053), NF-κB (sc-8008), β-tubulin (sc-47778), and goat anti-mouse IgG secondary antibodies (sc-2005) were obtained from Santa Cruz Biotechnology, Texas, USA. The enhanced chemiluminescence (ECL) (Trident femto Western HRP Substrate, Cat No. GTX14698) was obtained.

### Cell lines

The adenocarcinomic human alveolar basal epithelial cell line (A549) was obtained from American Tissue Culture Collection (ATCC). We used DMEM medium as cells grow medium, containing 10% FBS, 100 µg/mL streptomycin, and 2 mM L-glutamine 100 units/mL penicillin incubated at 5% CO_2_, 100% relative humidity, 95% air, and 37 ^ο^C in a humidified incubator. Observe the cell culture under an inverted microscope to evaluate the composition and amount of presence of bacterial and fungal contaminants.

### Cell viability assay

To show the cytotoxic effect of apocynin (1, 2, 4, 6, and 8 μM), the MTT assay was used to create the cell viability test (El-Shafey and Elsherbiny 2023). A tetrazolium salt called MTT was employed to study mitochondrial activity. The mitochondrial succinate dehydrogenase enzyme, which splits the tetrazolium ring and turns it into a purple formazan that is insoluble, accelerated the enzymatic reduction of MTT. In a 96-well plate, A549 cells were grown at a density of 5 × 10^3^ cells/well. In a 96-well plate, the cells were cultivated for 24 h in 200 μL of DMEM containing 10% FBS. Cells were treated with apocynin at several doses (1, 2, 4, 6, and 8 μM) for 48 h after the initial 24-h period. Following that, 15 μL of 5 mg/mL MTT was applied to each well in the plate containing the cells, and the plates were incubated for 4 h at 37 °C. Following the removal of DMEM, 100 μL of DMSO was applied to each well, and the microplate reader (μ-Quant, Epoch BioTek Instruments, Vermont, USA) was used to quantify the results at 590 nm. The control was DMEM devoid of samples.

$${\%}\;\text{Cell viability}= 100\;-\;\text{ Abs }(\text{sample})/\text{Abs }(\text{control}) \times 100$$

### Cell death detection of apoptosis

For the identification of cell death, to identify the kind of cell death brought on by varying AP doses in A549 cells, the ELISAPLUS (CDD) colorimetric apoptosis test was employed. From the cells that were trypsinized and sold into 96-well plates, 5 × 10^3^ were planted in each box with 100 μL of medium (3 replicates were made). It was kept in the incubator for 24 h. At the end of 24 h, the medium in the wells was removed, and then, 1, 2, 4, 6, and 8 μM concentrations of AP were applied. It was kept in the incubator for 24 and 48 h. The plate was centrifuged at 10 min 200 G, and the cell supernatant was carefully removed. The pellets formed at the bottom of the plate were suspended with 200 μL of lysis buffer, and the pellets were allowed to melt for 30 min at room temperature. The 96-well plate was centrifuged at 10 min at 200 G. Twenty microliters of the supernatant was carefully transferred to the plate from the Streptavidin-Coated kit without shaking the plate. Twenty microliters of each of the positive controls included in the kit, negative control (supernatants of A549 cell lysates without treatment), and blank (incubation buffer) were transferred to streptavidin-coated wells, with 20 μL corresponding to the middle of the wells. All wells received 80 μL of immunoreagent, the plate was covered with adhesive tape, and it was incubated for 2 h at room temperature while being mixed at 300 rpm in a shaker. After removing the solution from the wells with care at the end of the period, the wells were cleaned 3 times using 300 μL of incubation buffer. Each well received 100 μL of ABTS solution. The desired color formation was achieved by mixing in a shaker for 10–20 min at 250 rpm. All wells received 100 μL of ABTS Stop Solution at the end of the period, and an Epoch BioTek Take3 plate reader was used to read the results against wavelengths of 405 and 490 nm. It was agreed that the negative control’s enrichment factor was 1. Divide the mean absorbance value of the sample by the mean absorbance value of the negative control, calculated by subtracting the absorbance value of the blank from the total absorbance value (Apaydin Yildirim et al. 2024). The ratio of the number of mononucleosomes and oligonucleosomes in each sample to the number in the control is an appositive factor that is the enrichment factor, which was the relative absorbance value that was obtained.

### Cell lysate preparation

Cells were seeded into flasks for both the control and treatment groups, and AP was administered once the cells had adhered to the flask bottom. Allow the treated cells to incubate. At the end of this period, the control cells were collected for protein isolation. Prepare lysis buffer to separate proteins from the cell pellets obtained after centrifugation. Add 10 μL of orthovanadate sodium solution, 10 μL of phenylmethylsulfonyl fluoride, and 10 μL protease inhibitor per 1 mL of RIPA (sc-24948, Santa Cruz Biotechnology, Texas, USA) lysis buffer. Qiagen 0.2 mm diameter fine beads were placed in the Eppendorf wells of the Qiagen TissueLyser II device, which was pre-cooled at − 20 °C, and homogenization was performed at 50 rpm for 1 min. Then, add 600 μL of RIPA lysis buffer to all vessels, and disrupt cells in the affected tissue at 50 rpm for 1 min; this step was repeated twice. After this step, the tubes were centrifuged in a shaker block at 16,000 G, for 20 min (4 °C). The cell supernatants were transferred to new Eppendorf tubes and used in biochemical analyses (Yildirim et al. 2024).

### Cell lysate oxidative stress parameter analysis

The levels of ischemia-modified albumin in cell lysates were determined using the albumin cobalt-binding test as described by Bar-Or et al. (2001). Briefly, 50 µL of cell lysates were mixed with 200 µL of 0.1% cobalt chloride solution and incubated for 10 min at room temperature to allow albumin cobalt binding. Following this, 50 µL of dithiothreitol (1.5 mg/mL) was added, and the mixture was incubated for an additional 2 min. The reaction was stopped by adding 1.0 mL of 0.9% NaCl solution. The absorbance was measured at 470 nm using a spectrophotometer (Bar-Or et al. 2000). As this analysis was performed manually without commercial kits, inter- and intra-assay variation data were not calculated. However, to ensure precision, all samples were analyzed in duplicate, and consistent procedures were followed throughout the experiments. Advanced oxidation protein product (AOPP) measurement was done according to the method (Renault et al. 2006).

GSH levels were measured by Farnandez’s method (Ball 1966; Fernandez and Videla 1981). Lipid peroxidation levels were determined using the method described by Placer et al. (1966). The color produced by the interaction of malondialdehyde (MDA) with thiobarbituric acid at 532 nm was used to quantify the absorbance of the color to determine the amount of lipid peroxidation in cell lysates. SOD activity was measured using the method proposed by Sun et al. (1988). CAT activity was calculated using the Goth method, which quantifies the amount of hydrogen peroxide decomposed per unit time (Goth 1991). All absorbance values were quantified using an ELISA reader (BioTek μQuant MQX200/USA).

### Western blot analysis

Smith’s Thermo PierceTM BCA measurement kit was used to quantify the proteins after they were extracted from A549 cells that had been exposed to apocynin for 24 h. Ten percent of SDS-PAGE (sodium dodecyl sulfate-polyacrylamide gel electrophoresis) was used to load the produced cell samples. After being moved to the PVDF nitrocellulose membrane, the proteins were blocked for one and a half hours using 5% BSA. Following blocking, the membrane underwent 5-min TBST washes. The membranes that were incubated at + 4 °C for the entire night were probed with primary antibodies against Bax (sc-20067, Santa Cruz Biotechnology, Texas, USA), Bcl-2 (sc-7382, Santa Cruz Biotechnology, Texas, USA), caspase-3 (sc-56053, Santa Cruz Biotechnology, Texas, USA), NF-κB (sc-8008, Santa Cruz Biotechnology, Texas, USA), and β-tubulin (sc-47778, Santa Cruz Biotechnology, Texas, USA). Following a 5-min TBST wash, the blots were incubated for 2 h at room temperature with goat anti-mouse IgG secondary antibodies (sc-2005, Santa Cruz Biotechnology, Texas, USA). The membrane was rinsed 5 times with TBST for 5 min each after being incubated with the secondary antibodies. The enhanced chemiluminescence (ECL) (Trident femto Western HRP Substrate, Catalog No. GTX14698) was used in western blotting substrate to visualize protein bands, and the Bio-Rad Gel Doc XR Imaging System (Bıo-Rad, USA) was used to record the results. The ImageLab software from Bio-Rad was used to do a densitometric analysis of the bands. For every sample, at least three measurements were taken again (Dogan et al. 2024).

### Immunofluorescence staining method

The cultivated A549 cells were treated for 30 min in a paraformaldehyde solution. Cells were then maintained for 5 min in 3% H_2_O_2_. After washing the cells with PBS, a 0.1% Triton-X solution was added, and they were left for 15 min. Following the incubation period, the cells were covered with a protein block and left in the dark for 5 min. Following the usage instructions, the primary antibody (caspase-3 Cat No. sc-56053, Santa Cruz Biotechnology, Texas, USA; dilution ratio: 1/100 UK) was then dropped and incubated. Immunofluorescence secondary antibody (FITC Cat No. ab6785, diluent ratio: 1/500, UK) was used as a secondary marker and left without light for 45 min. After applying DAPI (Cat No. D1306, dilution ratio: 1/200 UK) to preparations and letting them be left without light for 5 min, the sections were covered with a coverslip. Analysis of the stained sections was done using a Zeiss AXIO GERMANY fluorescent microscope. The ZEISS Zen Imaging Software application was used to evaluate five randomly selected areas from each image in order to determine the level of positive staining from the immunofluorescence staining images. To statistically describe the data, the mean and standard deviation (mean ± SD) for area percentage were employed (Gursu et al. 2024).

### Statistical analysis

Statistical analyses were performed using the SPSS 22.0 package software program. Data were analyzed normality using the Shapiro-Wilk test and homogeneity of variance using Levene’s test. To compare statistical variations among different groups, Tukey’s test was combined with a one-way analysis of variance (ANOVA). The data was presented using a mean ± standard error of the mean (SEM) to showcase the information. When the *p*-value was less than 0.05, it was considered significant. A *p*-value of less than 0.05 was deemed statistically significant.

## Results

**Fig. 1:**
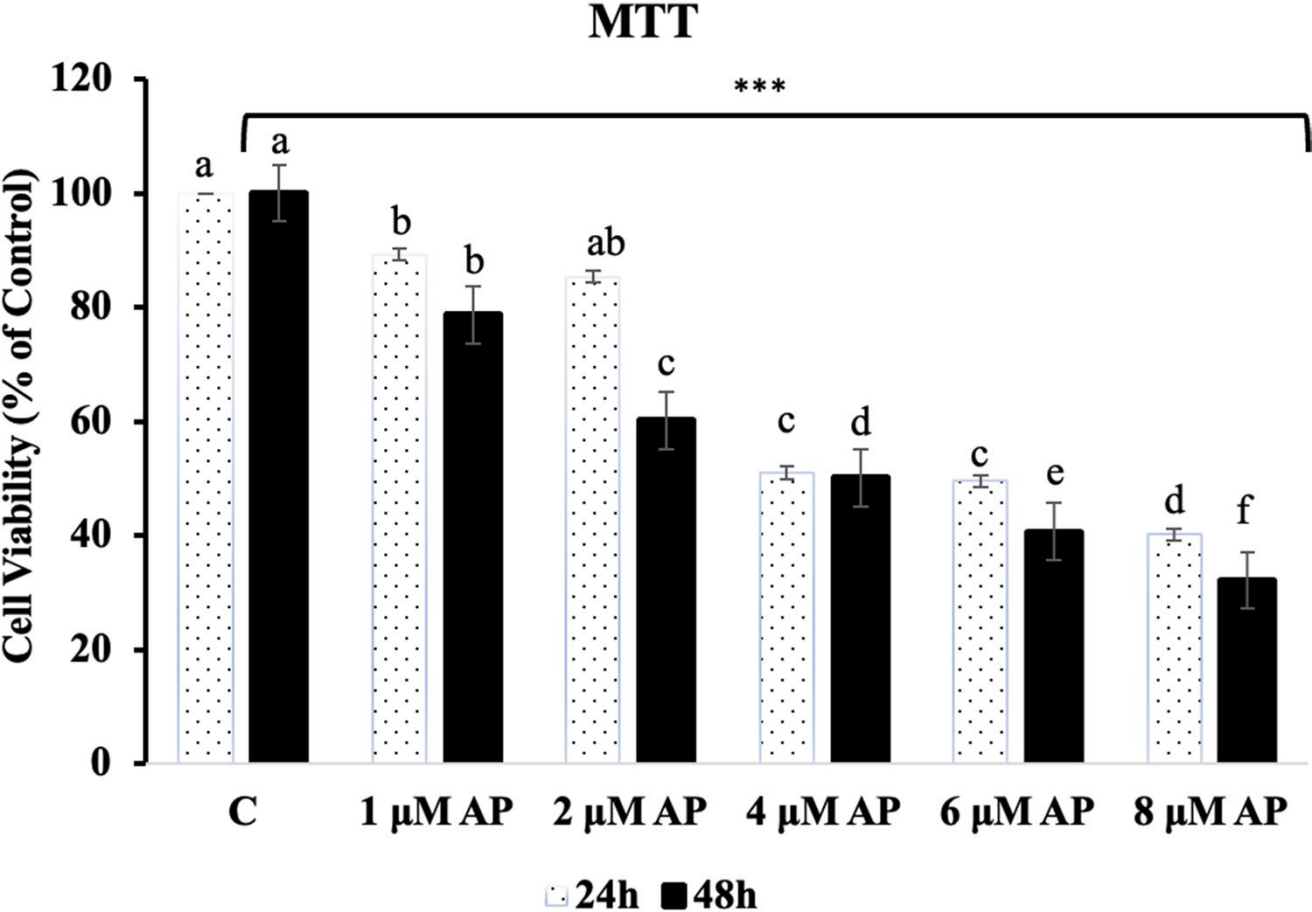
Percentage of cell viability on different concentrations of AP against A549 cells using MTT Assay. Data are expressed as mean ± SEM (***p < 0.001)

The mean of the number of viable cells in untreated control in A549 cell is taken as 100% viability for that cell, and accordingly, the mean of treated (24 and 48 h) cells at each dose was normalized for A549 cell. The result is shown in Fig. 1. AP treatment is evident from Fig. 1 that a comparatively higher effect was observed in A549 cells. MTT assay was used to quantify AP’s inhibitory effect on A549 cell proliferation. For 24 and 48 h, A549 cells were exposed to varying doses of AP. A549 cell growth curves were created, and the viability of the cells was assessed. A549 cell growth and proliferation were decreased by AP in a time and dose-dependent manner, as illustrated in Fig. 1 (*p* < 0.001). These findings suggested that AP could stop A549 cells from proliferating for extended periods of time and at larger concentrations.

**Fig. 2:**
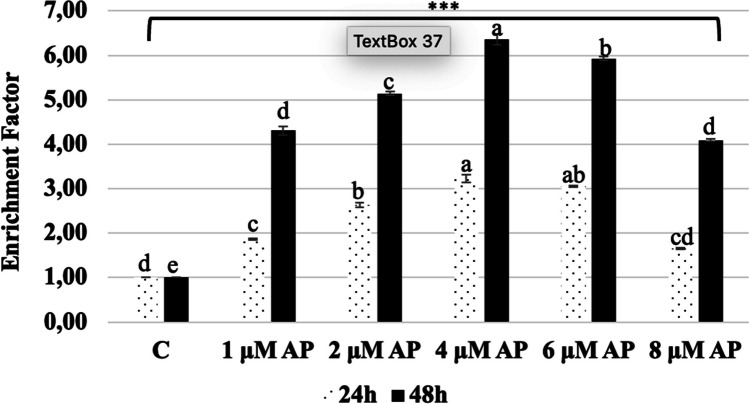
Apoptotic effect of AP at 24 and 48 h by CDD ELISA test in A549 cells. Data are expressed as mean ± SEM (***p < 0.001). The rate of apoptosis is reflected by the nucleosomes in the cytoplasm, shown along the *y*-axis. Experiments were performed in triplicate and repeated three times

Apoptosis is one of the important factors confirming cellular damage. Biochemically, one hallmark of apoptosis, namely, DNA fragmentation in the nucleus, was measured to determine if the AP would prevent or enhance apoptosis in A549 cells. Figure 2 shows the apoptotic effect of AP at 24 and 48 h by CDD ELISA test in A549 cells. AP treatment has induced apoptosis in A549 cells, as shown by the increase in DNA fragmentations in the cell lysate. Figure 2 shows that Ap treatment significantly increased the amount of DNA fragments in the A549 cells 1 (*p* < 0.001).

We evaluated the activity of antioxidant enzymes in terms of oxidative damage. As shown in Tables 1 and 2, AP, IMA, AOPP, and MDA levels in the supernatant of A549 cells after stimulation for 24 and 48 h were significantly increased in the AP treatment group compared to the control group (*p* < 0.001). GSH level and SOD and CAT activities were suppressed by AP at 24 and 48 h following treatment.

**Table 1:** Some biochemical parameters in the A549 cell supernatants following treatment with AP for 24 h

	İMA (ABSU)	AOPP (μmol/L)	GSH (mmol/L)	MDA (mmol/L)	SOD (U/mL)	CAT (kU/L )	*P*
C	1.11 ± 0.03^f^	0.09 ± 0.00^c^	1.20 ± 0.00^a^	10.15 ± 0.07^c^	18.58 ± 0.15^a^	131.16 ± 0.40^a^	.000
1 μM AP	1.18 ± 0.01^ef^	0.10 ± 0.0^b^	1.18 ± 0.01^a^	11.10 ± 0.26^b^	17.16 ± 0.10^b^	131.10 ± 0.44^a^	.000
2 μM AP	1.24 ± 0.02^de^	0.10 ± 0.0^b^	1.13 ± 0.01^b^	11.35 ± 0.26^b^	17.60 ± 0,11^b^	129.30 ± 0.38^b^	.000
4 μM AP	1.62 ± 0.01^a^	0.18 ± 0.0^a^	0.94 ± 0.01^d^	17.80 ± 0.09^a^	10.27 ± 0.08^e^	94.61 ± 0.52^e^	.000
6 μM AP	1.33 ± 0.01^b^	0.11 ± 0.0^bc^	1.07 ± 0.02^c^	11.95 ± 0.33^b^	13.03 ± 0.43^d^	123.70 ± 0.33^d^	.000
8 μM AP	1.26 ± 0.01^cd^	0.10 ± 0.0^bc^	1.19 ± 0.00^a^	11.38 ± 0.14^b^	14.24 ± 0.14^c^	125.53 ± 0.29^c^	.000

*C*, Control; *AP*, Apocynin. Data of all group were expressed as mean ±standard error (SEM). ***P < 0.001; ^a, b, c, d, e, f^ The difference between the averages shown with different letters in the same column is significant

**Table 2:** Some biochemical parameters in the A549 cell supernatants following treatment with AP for 24 h

	İMA (ABSU)	AOPP (μmol/L)	GSH (mmol/L)	MDA (mmol/L)	SOD (U/mL)	CAT (kU/L )	*P*
C	1.00 ± 0.05^d^	0.09 ± 0.00^c^	1.18 ± 0.00^a^	8.90 ± 0.30^c^	24.24 ± 0.20^a^	132.81 ± 1.45^a^	.000
1 μM AP	1.00 ± 0.05^d^	0.09 ± 0.00^bc^	1.18 ± 0.00^a^	8.90 ± 0.30^c^	24.24 ± 0.20^a^	132.81 ± 1.45^a^	.000
2 μM AP	1.23 ± 0.02^c^	0.09 ± 0.00^c^	1.16 ± 0.01^ab^	9.55 ± 0.25^bc^	23.10 ± 0.34^b^	130.02 ± 0.18^bc^	.000
4 μM AP	1.66 ± 0.02^a^	0.19 ± 0.00^a^	1.09 ± 0.00^d^	17.70 ± 0.31^a^	10.49 ± 0.19^e^	94.29 ± 0.02^d^	.000
6 μM AP	1.36 ± 0.02^b^	0.10 ± 0.00^b^	1.12 ± 0.00^cd^	10.17 ± 0.10^b^	16.50 ± 0.25^d^	127.83 ± 0.41^c^	**.**000
8 μM AP	1.27 ± 0.03^bc^	0.09 ± 0.00^c^	1.14 ± 0.01^bc^	9.72 ± 0.23^bc^	18.91 ± 0.20^c^	129.89 ± 0.14^bc^	.000

*C*, Control; *AP*, Apocynin. Data of all group were expressed as mean ± standard error (SEM). ***P < 0.001; ^a, b, c, d, e^ The difference between the averages shown with different letters in the same column is significant

**Fig. 3:**
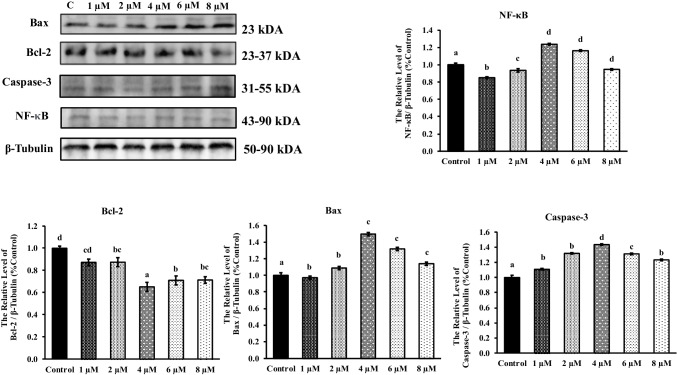
Effects of AP on A549 cell line levels of Bcl-2, Bax, caspase-3, and NF-κB genes at 24 h. The bar graph is shown as mean ± standard error (SEM) (*p* < 0.001). Relative band intensities of the proteins are measured with ImageJ software. Data are expressed as mean ± SEM (*n* = 3), *p < 0.05 vs. respective control (untreated cells)

**Fig. 4:**
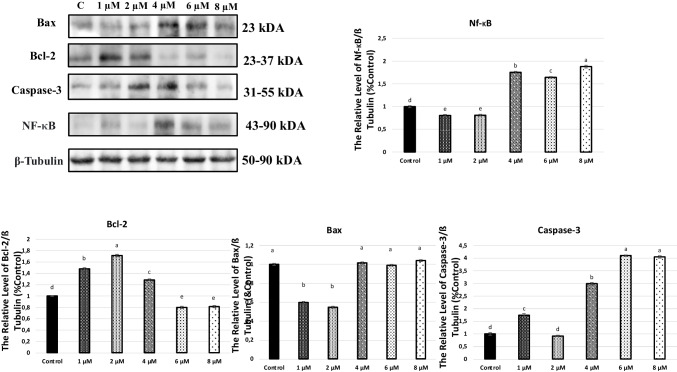
Effects of AP on A549 cell line levels of Bcl-2, Bax, caspase-3, and NF-κB genes at 48 h. The bar graph is shown as mean ± standard error (SEM) (*p* < 0.001). Relative band intensities of the proteins are measured with ImageJ software. Data are expressed as mean ± SEM (*n* = 3), *p < 0.05 vs. respective control (untreated cells)

In AP-exposed A549 cells, pro-apoptotic protein levels were assayed at 24 and 48 h by western blotting (Figs. 3 and 4). Treatment with AP significantly reduced Bcl-2 protein expression at 24 and 48 h after AP treatment significantly suppressed Bcl-2 expression compared to the group without AP treatment in A549 cells. The NF-κB pathway is an important signaling pathway that regulates the expression of inflammatory mediators. As shown in Figs. 3 and 4, NF-κB protein expression was significantly increased in the 4, 6, and 8 μM AP treatment group compared to the control group 24 and 48 h (*p* <0.001). On exposure to 4, 6, and 8 μM concentrations of AP, elevated caspase-3 levels in A549 cells were observed. In AP (4, 6, and 8 μM)-exposed A549 cells, the caspase-3 protein expression level was also higher relative to untreated cells. Treatment with AP significantly increased Bax, NF-κB, and caspase-3 protein expressions at 24 and 48 h after AP treatment (Figs. 3 and 4) (*p* < 0.001). The results showed that AP could significantly inhibit the proliferation of lung cancer cells in vitro and induce cell apoptosis. This antitumor activity may be achieved by downregulating Bcl-2 and upregulating Bax, suggesting that AP can be used as a potential chemical drug for the clinical treatment of lung cancer.

**Fig. 5:**
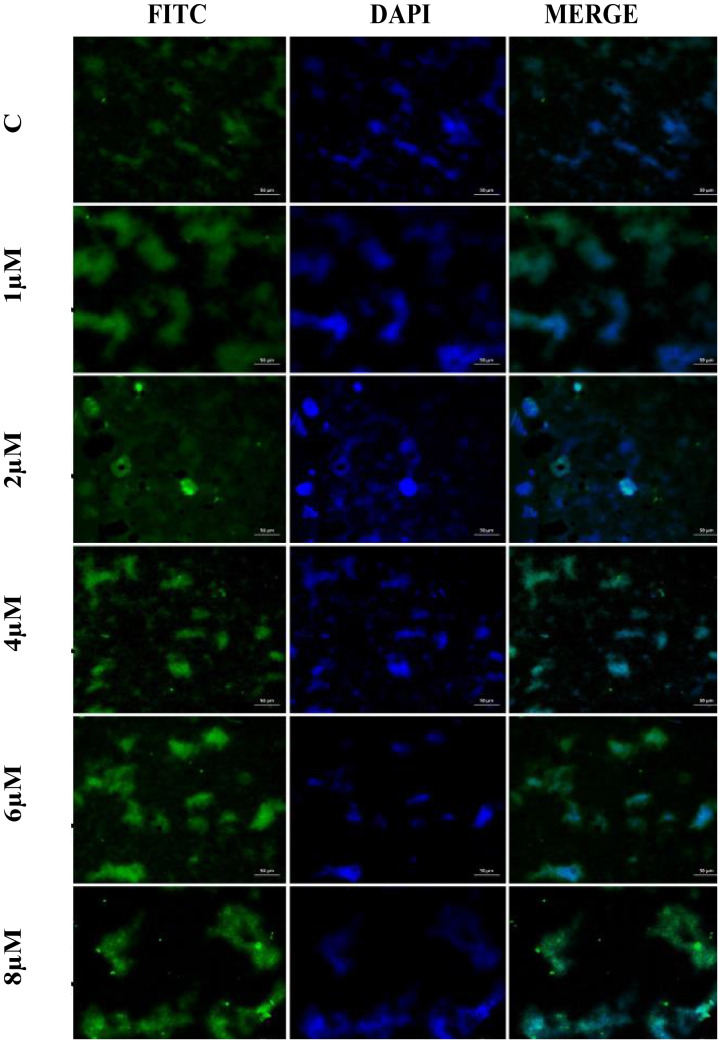
Apocynin A549 cell line 24 h; cytoplasmic caspase-3 expressions in A549 cells (FITC), IF, Bar: 50 µm, × 40

**Fig. 6:**
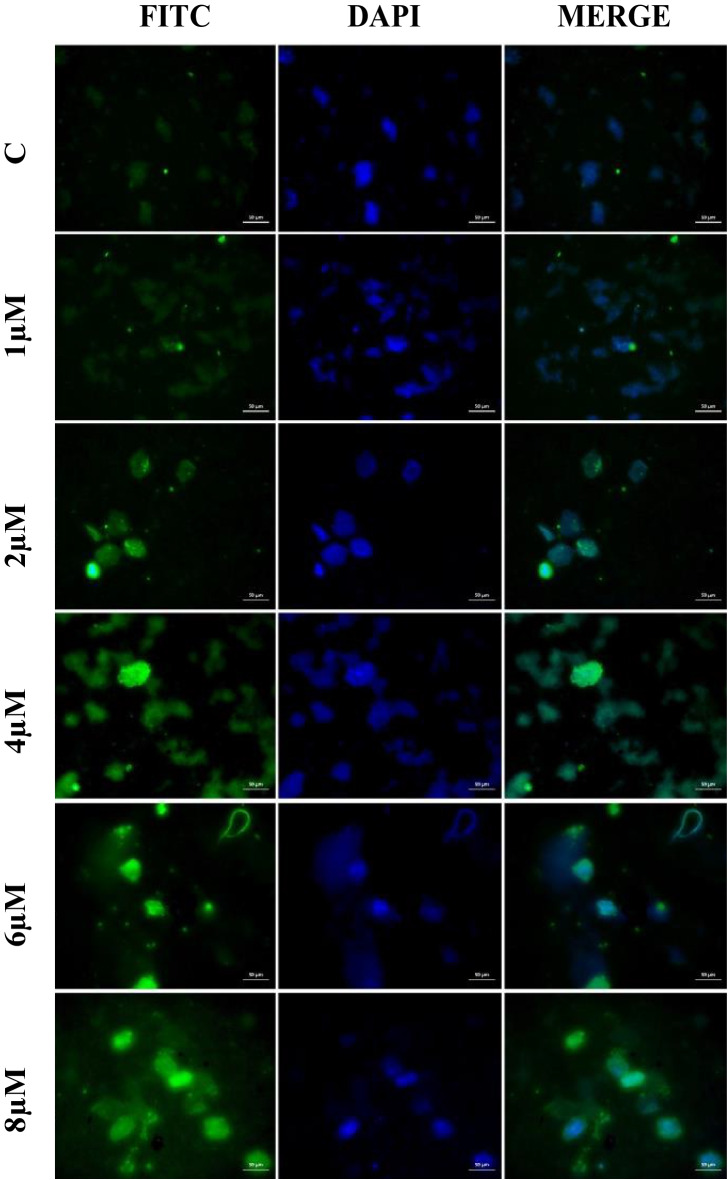
Apocynin A549 cell line 48 h; cytoplasmic caspase-3 expressions in A549 cells (FITC), IF, Bar: 50 µm, × 40

**Fig. 7:**
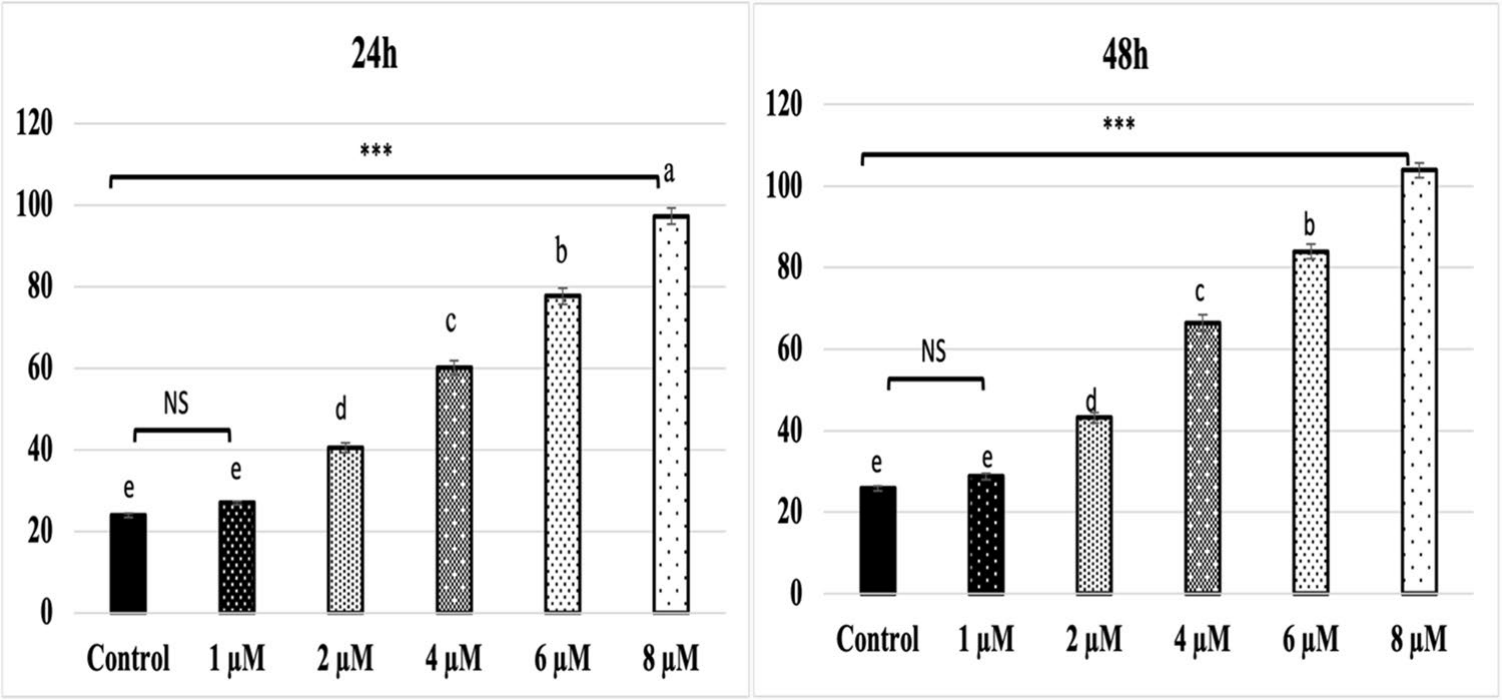
Apocynin A549 cell line 24 and 48 h immunofluorescence staining data and statistical analysis results. Control: A549 cell line, 1–8 µM AP treatment groups. Data are expressed as mean ± SEM (****p < 0.0001, NS: non-significant *p* > 0.05)

The immunofluorescence staining results of the effects of Apocynin on A549 cells for both 24 h and 48 h are the same results, and the images and graphs are shown in Figs. 5 and 6. As a result of immunofluorescence staining performed on the control group A549 cell line, very mild intracytoplasmic caspase-3 expressions were observed in the cells. Immunofluorescence staining was performed on the A549 cell line in the group treated with 1 μM AP, very mild intracytoplasmic caspase-3 expressions were detected in the cells; 2 μM AP, mild intracytoplasmic caspase-3 expressions in the cells; 4 μM AP, moderate intracytoplasmic caspase-3 expressions in the cells; 6 μM AP, severe intracytoplasmic caspase-3 expressions in the cells; and 8 μM AP, very intense intracytoplasmic caspase-3 expressions in the cells. Data of immunofluorescence staining findings and statistical analysis results are shown in Fig. 7.

## Discussion

Although the beneficial antioxidant, anti-apoptotic, and anti‐inflammatory effects of AP have been reported in inflammatory airway and lung diseases such as chronic obstructive pulmonary disease, lung fibrosis, and asthma, there is no literature on its antiproliferative, antioxidant, and apoptotic effects on the A549 cell line in vitro, so the current study evaluates the effectiveness of AP in the prevention and treatment of lung cancer.

Biologically active substances can induce cancer cell apoptosis by increasing ROS levels (Prasad et al. 2017). An imbalance between the pro-oxidative state and the cell’s antioxidant defenses elevates ROS levels, which can oxidize phospholipids in the cellular membrane, forming malondialdehyde (MDA), and a mutagenic product of lipid peroxidation. This oxidative damage leads to cellular dysfunction and apoptosis (Tsikas 2017). Superoxide dismutase (SOD) is a key enzyme responsible for eliminating ROS. Human cells are rich in SOD1 (Cu/Zn-SOD), making SOD1 detection a reliable indicator of SOD activity. Increased SOD levels in A549 cells can reduce ROS-induced apoptosis, whereas reducing SOD activity in cancer cells promotes apoptosis (In et al. 2016). Similarly, catalase (CAT) decomposes hydrogen peroxide in cancer cells, preventing the formation of toxic free radicals and thereby inhibiting apoptosis (Xiao et al. 2015).

Apocynin effectively and selectively inhibits NOX in activated leukocytes. It is a natural substance that prevents ROS production by inhibiting methoxy-substituted catechol (Stefanska et al., 2008). Several cell culture and animal studies have shown that AP can reduce neutrophil oxidative burst and neutrophil chemotaxis thereby reducing neutrophil-mediated cell damage (Impellizzeri et al. 2011). AP reduces lipid peroxidation and inhibits NOX expression and NOX activity. Apocynin has been reported to exhibit selective cytotoxicity against lung cancer cells rather than normal lung fibroblast cells, suggesting its potential as a therapeutic agent for lung cancer treatment (Paul et al. 2020). In this study, AP was shown to increase ROS levels in A549 cells, promoting apoptosis by raising MDA content while reducing SOD and CAT activities.

Apoptosis is triggered by a series of physiological and pathological signals. These signals regulate death-related genes, activating pathways such as the death receptor pathway, which involves Bcl-2, Bax, and the caspase pathway (Liener et al. 2003; Xiao et al. 2017). Bcl-2 is an upstream regulator of the apoptotic pathway. It can form heterodimers with the pro-apoptotic Bax family proteins, neutralizing their pro-apoptotic effects and thereby inhibiting cell apoptosis (Kim et al. 2003).

Paul et al. (2020) observed a significant increase in Bax and cleaved caspase-3 expression, along with a decrease in Bcl-2 expression, in AP-treated cells. However, in cells treated with 1 mM 3-MA, the effects of AP treatment were attenuated after 48 h compared to control cells (Paul et al. 2020). To further investigate the mechanism, we analyzed the expression of Bax, Bcl-2, caspase-3, and Nf-κB proteins using western blot. Bcl-2 and Bax are key regulators of the apoptotic pathway, with Bcl-2 family members altering mitochondrial membrane permeability and initiating caspase activation, ultimately leading to apoptosis.

NF-κB family proteins are well-established candidates in tumor progression and angiogenesis, making them important targets for cancer therapy. Aberrant expression of NF-κB has been observed in many tumors, and several inhibitors are currently in clinical trials for cancers, including lung cancer (Rasmi et al. 2020). In the cytoplasm, NF-κB is inactive as it forms a complex with its inhibitor protein, IkB. Upon activation of IkB kinase via phosphorylation, IkB undergoes proteasomal degradation, releasing the NF-κB heterodimer. The free NF-κB then translocates to the nucleus, where it triggers the transcriptional upregulation of target genes depending on the regulatory sequences in the genome (Albensi 2019). There are in vivo studies that have found that it suppresses the Nf-κB pathway and subsequent transcription of proinflammatory cytokines in lung tissue, thereby alleviating lung injury (Kim et al. 2012; Choi et al. 2017; Adams and Cory 1998).

Interestingly, NF-κB can have pro-apoptotic effects under certain conditions, such as DNA damage, hypoxia, or serum deprivation, by downregulating anti-apoptotic genes (Campbell et al. 2004; Ryan et al. 2000). It may also promote apoptosis through the activation of pro-apoptotic genes. Inhibition of NF-κB has been shown to induce apoptosis, reduce cell proliferation, and suppress metastasis (Perkins 2012; Sun et al. 2021). Thus, targeting NF-κB acts as a double-edged sword—it can induce apoptosis while also reducing metastasis. This version improves flow, reduces redundancy, and enhances readability while preserving the key points. It has been reported that apocynin can primarily inhibit cancer progression in various cancers by blocking the PI3K-AKT-GSK3β axis and downregulating cell cycle proteins, as well as reducing Ki-67 protein expression in tumors (Kato et al. 2015; Jantaree et al. 2017).

AP inhibited apoptosis by reducing the expression of the anti-apoptotic protein Bcl-2 and enhancing the expression of pro-apoptotic proteins Bax, Nf-κB, and caspase-3. Thus, we concluded that the anti-apoptotic effect of AP may be mediated through the downregulation of Bcl-2 and upregulation of caspase-3. In our experiment, we used CDD ELISAPLUS colorimetric detection of apoptosis, western blotting, and immunofluorescence staining to evaluate apoptosis of A549 cells and found that AP protects against A549 cell injury.

This study examined the effects of AP on the proliferation and apoptosis of A549 lung cancer cells in vitro, as well as the expression of apoptosis-related factors following AP treatment. The results demonstrated that AP significantly inhibited the proliferation of lung cancer cells and induced apoptosis. This antitumor activity appears to be mediated by the downregulation of Bcl-2 and upregulation of Bax, indicating that AP has potential as a chemical agent for the clinical treatment of lung cancer. However, further research, including clinical trials, is required to evaluate the long-term effects of AP, and its mechanisms should be explored in animal models.

## Conclusion

In conclusion, oxidative stress plays a significant role in various diseases, particularly inflammatory conditions. The findings of this study suggest that targeting NOX may hold therapeutic potential for lung cancer, with AP emerging as a promising candidate. However, further comprehensive studies are required to explore this topic in depth. Notably, this report is the first to demonstrate that AP exerts an anti-apoptotic effect by regulating the expression of Bax, Bcl-2, Nf-κB, and caspase-3 expression in vitro.

## Data Availability

All source data for this work (or generated in this study) are available upon reasonable request.
